# Inhalation Anesthesia with Sevoflurane during Intravitreal Bevacizumab Injection in Infants with Retinopathy of Prematurity

**DOI:** 10.1155/2013/435387

**Published:** 2013-06-06

**Authors:** Orhan Tokgöz, Alparslan Şahin, Adnan Tüfek, Yasin Çınar, Abdülmenap Güzel, Taner Çiftçi, Feyzi Çelik, Harun Yüksel

**Affiliations:** ^1^Department of Anesthesiology, Faculty of Medicine, Dicle University, 21280 Diyarbakır, Turkey; ^2^Department of Ophthalmology, Faculty of Medicine, Dicle University, 21280 Diyarbakır, Turkey

## Abstract

*Bacground*. To investigate the anesthetic management in premature infants with retinopathy of prematurity (ROP) treated with intravitreal bevacizumab (IVB) injections. *Methods*. A retrospective chart review was performed for the patients with ROP who had IVB injection. Clinical characteristics, demographic variables, anesthetic medications, operation techniques, time intervals, and complications were recorded. *Results*. Sixty-six eyes of 33 patients (23 males, 10 females) with type 1 ROP who were treated with IVB were included. A total of 66 anesthetic applications were performed. Mean gestational age at birth was 28.3 weeks (range 25–33). Mean birth weight was 1300 g (range 600–1850). Serious ocular and systemic complications were not observed in any infant. *Conclusion*. Inhalation anesthesia with sevoflurane during IVB treatment in premature infants with ROP may be appropriate for anesthetic management.

## 1. Introduction

Retinopathy of prematurity (ROP) is a retinal vascular disease in premature infants, and all babies less than 32 weeks gestational age or less than 1501 g birth weight should be screened for ROP [1]. ROP leads to blindness when its treatment is not on time or effective. Improvements in neonatal intensive care have increased the survival rates of premature infants. Thus, the infants who need to be treated have been increasing [2].

Currently, laser photocoagulation (LPC) is the main treatment approach in ROP [3]. Besides a novel but off-label intravitreal injection of bevacizumab (IVB) has became a promising treatment alternative to LPC [4, 5]. IVB injection is a painful procedure for premature infants. Painful stimuli may cause undesired movement of the infants. IVB injection may have also potential risk for complications such as cataract formation and inappropriate injection site, if performed under topical anesthesia. Therefore, an ideal anesthesia management should be defined in infants treated with IVB. Sevoflurane is an inhalation anesthetic which enables rapid induction and recovery and is widely used in infants [6].

To our knowledge, sevoflurane mask anesthesia in ROP patients treated with IVB has not been studied before. The present study is therefore designed to evaluate the use of sevoflurane to achieve sedation for intravitreal injection of bevacizumab in nonintubated infants with ROP.

## 2. Materials and Methods

The Institutional Review Board approval was obtained from the Ethical Committee of Dicle University, and the study was conducted in compliance with the Declaration of Helsinki. The medical records of patients with type 1 ROP who received IVB monotherapy were reviewed. Type 1 ROP was defined according to Early Treatment of Retinopathy of Prematurity Study results as follows: any stage of ROP with plus disease in zone 1; stage 2 or 3 ROP with plus disease in zone 2; or stage 3 ROP without plus disease in zone 1 [7].

Birth weight, gestational age at birth, IVB injection time, operation time, recovery time, comorbidities, and complications were recorded. Before IVB injection, the parents or legal guardians of the infants had been informed about the procedure. After informed consent was obtained for IVB injection, the injections were performed at the operation room.

An ophthalmologist, pediatrician, and anesthesiologist had examined all patients preoperatively. The infants fasted at least 4 hours prior to the injection. The patients were taken to the operation room with a spontaneous respiration and monitored with pulse oximetry, electrocardiogram, and noninvasive arterial blood pressure. The pupil was dilated with 0.5% tropicamide (Tropicamide, Bilim, Turkey) and 2.5% phenylephrine (Mydfrin, Alcon, USA) before intravitreal injection. Anesthesia had been induced with 5% sevoflurane in 100% oxygen until the loss of consciousness, and then maintained with 2% sevoflurane in 50% oxygen. The time to loss of consciousness (absence of crying or movement) was recorded as induction time. Patients spontaneously breathed with face mask sevoflurane throughout the procedure. Under sterile conditions, an eyelid speculum (Katena, Denville, New Jersey, USA) was inserted and the eyeball was deviated to inferior nasal by a scleral depressor. Bevacizumab (Altuzan, Roche, İstanbul, Turkey) with a dose of 0.625 mg (0.025 mL) was injected intravitreally 1mm posterior to the superior temporal limbus via 30-gauge 2 needle. An experienced ophthalmologist performed all IVB injections. Simultaneously with the IVB injection, sevoflurane anesthesia was terminated and the circuit was flushed with 100% oxygen.

At the end of surgical procedure, patients were observed until spontaneous eye opening and/or crying occurred. After recovery, all patients were transferred to the newborn unit in a preheated transport incubator. Feeding was prohibited within 2 h after recovery.

The duration of the anesthesia (from the beginning of induction of sevoflurane inhalation to spontaneous movement and/or crying), the induction time, respiratory depression (SpO_2_ < 95%), and recovery time (from the end of the sevoflurane inhalation to spontaneous movement and/or crying) were recorded. Any adverse event in the recovery period and in feeding was recorded until discharged home. The fellow eyes of the patients were treated from 5 to 7 days after IVB treatment of the primary eyes. 

Statistical analyses were performed using the SPSS software version 11.5 (SPSS Inc., Chicago, IL, USA). Data were expressed as mean ± SD, minimum–maximum, or %. Induction and recovery times and anesthesia duration during first and second IVB administrations were compared with Wilcoxon rank test. A *P* value less than 0.05 was considered significant.

## 3. Results

Demographical data such as gestational age and weight at birth and at the time of the treatments are shown in Table 1. There were 33 infants (23 males, 10 females) in the study. Mean gestational age at birth was 28.3 weeks (range 25–33). Mean birth weight was 1300 ± 45 g (range 600–1850). Six of the infants had respiratory distress syndrome, two had hydrocephalus, one had necrotizing enterocolitis, and one had hypoxic ischemic encephalopathy.

A total of 66 anesthetic applications were performed without any complication. Neither severe apnea resulting in desaturation nor bradycardia due to apnea during the perioperative course was observed. Each eye of the infants was treated with a separate procedure. The average recovery time was 3.9 ± 0.9 min in all infants.

During recovery, three patients had sinus tachycardia and one patient had sinus bradycardia. Only two babies had apnea attacks which were treated with mask ventilation. No coughing or vomiting was observed during feeding after two hours of anesthetic recovery. Comparison of induction, recovery time, and anesthesia duration during the first and second IVB administrations showed no significant differences (Table 2).

## 4. Discussion

In the present study, sevoflurane mask anesthesia was performed smoothly, which facilitated the injection of intravitreal bevacizumab in patients with ROP. Endotracheal intubation was not needed in any of the infants. There was no respiratory depression or coughing during postanesthetic recovery. 

Bevacizumab, which is an antivascular endothelial growth factor (VEGF) antibody, is used in the treatment of ocular neovascular disorders including ROP [8]. Although anti-VEGF treatments in ROP are offlabel, it allows vascularization of the avascular retina. Relatively short procedure time with milder stress is an advantage of intravitreal bevacizumab injection compared to LPC.

The appropriate anesthetic management for anti-VEGF therapy in patients with ROP is one of the issues that was not well defined. Short anesthesia induction time, stability and controllability of anesthesia during examination, rapid recovery from anesthesia, and no residual anesthesia before discharge should be the main properties of a good anesthesia. As is known, sevoflurane is one of the best inhalation anesthetic that does not have any irritation in the respiratory tract, and it has small distribution coefficient, short induction and recovery times due its low blood gas partition coefficient, short equilibrium time, and quick induction making it a good choice for day surgery [9]. Selecting sevoflurane as a mask anesthetic agent provides fast depth of anesthesia and fast recovery without any prolonged effect. To the best of our knowledge, this is the first study conducted on IVB via sevoflurane mask anesthesia in ROP patients.

IVB treatment in ROP is stressful and painful to the neonate. This is partly because of the speculum used to keep the eyelids open and the intensity of the ophthalmoscope light [10]. Intravitreal injections may cause a short-term transient rise in intraocular pressure, which may result trigemino-vagal reflex [11]. Although topical anesthetic pretreatment can reduce the pain response [12] and general anesthesia is not necessary in all patients, topical anesthesia has some disadvantages such as stabilization of the eyeball during intravitreal injection. Because of the IVB injection under topical anesthesia in infants, inadvertent localization and possibility of lens damage may lead to complications. Immobility of the eye is very important during IVB since it has the potential complication of lens trauma [13]. Maneuvers that were used for eye immobility may induce stress in infants, and even examination of the eye under topical anesthesia was reported as an uncomfortable procedure [14]. Anesthesia should provide ideal surgical situation for the surgeon beside blockage of pain perception. 

Sevoflurane mask anesthesia was safely and effectively used in fundus examination of preterm infants [15]. We suggested that sevoflurane mask anesthesia might be a safe and simple method in IVB treatment of premature babies with ROP. 

The sevoflurane mask anesthesia has potential complications of general anesthesia such as laryngospasm, bronchospasm, and aspiration. Also the anesthetic management takes much time and has a recovery period. These factors may cause increasing burden of anesthetic resources. Although we did not experience such these complications, this is the negative aspect of our study.

In our clinical approach, we never used positive ventilation that may increase the risk of aspiration in these infants. Thus, the disadvantages of general anesthesia were not expected in such shorter procedures. Sevoflurane mask anesthesia causes minimal irritation due to preserved spontaneous ventilation and lower airway reflex stimulation. A survey from England showed that sedation and analgesia were always used in the treatment of ROP beside topical anesthetics [16]. Using premature infant pain profile, Cogen et al. found that 65% of the examinations without anesthesia were painful, compared with 27% with anesthesia in ROP examination [17]. In a similar study, 77.8% of the infants had mild pain during intravitreal injections under topical anesthesia [18]. The most important reason was the insertion of the lid speculum. Topical anesthesia did not prevent pain perception during lid speculum insertion. 

In conclusion, sevoflurane mask anesthesia is a good alternative in IVB injection for ROP without the associated perioperative ocular and anesthetic complications. Further studies with larger numbers of patients are needed to clarify the efficiency and safety of sevoflurane mask anesthesia in IVB injection for ROP.

## Figures and Tables

**Table 1:** Clinical characteristics of premature infants undergoing intravitreal injections for retinopathy of prematurity under sevoflurane mask anesthesia.

	*n*, Mean ± SD	Minimum–Maximum
Sex (male/female)	23/10	—
Age at the birth (gestational week)	28.3 ± 2.2	25–33
Weight at the birth (gram)	1300.5 ± 290.7	600–1850
Age at the first IVB (gestational week)	36.2 ± 3.5	31–44
Weight at the first IVB (gram)	2148.5 ± 450.3	1310–3130

IVB: Intravitreal bevacizumab.

**Table 2:** Comparison of induction time, recovery time, and anesthesia duration during the first and the second IVB administrations.

	First IVB	Second IVB	*P*
Anesthesia induction time (min)	2.6 ± 0.7	2.5 ± 0.8	NS
Recovery time (min)	3.9 ± 0.9	3.8 ± 1.1	NS
Anesthesia duration (min)	10.2 ± 1.7	9.9 ± 1.9	NS

IVB: Intra vitreal bevacizumab, NS: not significant.
